# Post-therapeutic microRNA-146a in liquid biopsies may determine prognosis in metastatic gastrointestinal cancer patients receiving ^90^Y-radioembolization

**DOI:** 10.1007/s00432-023-05185-0

**Published:** 2023-07-19

**Authors:** Heidrun Hirner-Eppeneder, Elif Öcal, Matthias Stechele, Osman Öcal, Sijing Gu, Melanie A. Kimm, Moritz Wildgruber, Lukas Salvermoser, Philipp Kazmierczak, Stefanie Corradini, Martina Rudelius, Guido Piontek, Maciej Pech, S. Nahum Goldberg, Jens Ricke, Marianna Alunni-Fabbroni

**Affiliations:** 1 Department of Radiology, LMU University Hospital, LMU Munich, Marchioninistraße 15, 81377 Munich, Germany; 2 Department of Radiation Oncology, LMU University Hospital, LMU Munich, Munich, Germany; 3 Department of Pathology, LMU University Hospital, LMU Munich, Munich, Germany; 4 Department of Radiology and Nuclear Medicine, University of Magdeburg, Magdeburg, Germany; 5 Goldyne Savad Institute of Gene Therapy and Division of Image-Guided Therapy and Interventional Oncology, Department of Radiology, Hadassah Hebrew University Medical Center, Jerusalem, Israel

**Keywords:** Radioembolization, Metastatic liver disease, microRNA, Liquid biopsy, Biomarker

## Abstract

**Purpose:**

The role of microRNA-146a (miR-146a) in defining the tumor immune microenvironment (TIME) is well established. The aim of this study was to evaluate circulating miR-146a as an early prognostic marker of ^90^Y-radioembolization (^90^Y-RE) in metastatic liver cancer and to assess the correlation between circulating miR-146a and TIME cellular composition in distant, yet untreated metastases.

**Methods:**

Twenty-one patients with bilobar liver lesions from gastro-intestinal cancer underwent lobar ^90^Y-RE. Biopsy of contralateral lobe abscopal tumors was acquired at the onset of a second treatment session at a median of 21 days after initial RE, immediately prior to ablation therapy of the contralateral lobe tumor. miR-146a was measured by RT-qPCR in plasma collected 24 h before (T1) and 48 h after (T2) initial unilobar ^90^Y-RE. The level of miR-146a was correlated with the infiltration of CD4 + , CD8 + , FoxP3 T cells, CD163 + M2 macrophages and immune-exhausted T cells in the abscopal tumor tissue acquired before the second treatment session.

**Results:**

Plasma samples collected at T2 showed a higher concentration of miR-146a with respect to T1 in 43% of the patients (*p* = 0.002). In these patients, tumors revealed a pro-tumorigenic immune composition with enrichment of Tim3 + immune exhausted cells (*p* = 0.021), in combination with a higher infiltration of CD163 + M2 macrophages and a lower infiltration of CD8 + T cells. Patients with a higher level of miR-146a after ^90^Y-RE showed a trend to shorter OS (*p* = 0.055).

**Conclusion:**

miR-146a may represent a novel prognostic biomarker for ^90^Y-radioembolization in metastatic liver cancer.

**Supplementary Information:**

The online version contains supplementary material available at 10.1007/s00432-023-05185-0.

## Introduction

In patients with liver metastases (metastatic liver cancer, mLC), loco-regional treatment by transarterial yttrium-90 (^90^Y)-radioembolization (RE) is an established treatment (Vogel et al. 2021; Reig et al. 2021; Helmberger et al. 2021; Karanicolas et al. 2021).^90^Y-RE has proven to be effective both in first (Salem et al. 2016) and second-line settings (Mulcahy et al. 2021), being able to boost immune response and indirectly to modulate the tumor immune microenvironment (TIME) composition in distant, non-treated yet, metastases. However, clinical outcomes vary, with a significant proportion of patients not achieving durable responses (Lau et al. 2013; Ricke et al. 2019). In addition, the low tolerance of the liver to irradiation, characterized by a rapid deterioration of hepatic function, can be a limiting factor (Sangro et al. 2008; Seidensticker et al. 2021; Ricke et al. 2021). Therefore biomarkers for treatment efficacy prediction are a highly needed tool. In the context of liquid biopsy, microRNAs (miRNAs) occupy a prominent role as non-invasive biomarkers in several types of diseases, including cancer. miRNAs are highly conserved, ~ 21–25 nucleotides long, non-coding RNA released from cells by active secretion (Kosaka et al. 2010) or after cell death (Turchinovich and Cho 2014). They are post-transcriptional repressors, inhibiting translation through transcript degradation (Di Leva et al. 2014; Hanahan and Weinberg 2000). Several studies, mainly in vitro and in animal models, have evaluated the modification of miRNA levels in response to radiation, confirming miRNAs as useful biomarkers for radio sensitivity (Drula et al. 2020). miRNAs play an important function in forging the tumor immune microenvironment (TIME), modulating the infiltration of immune cells and their function (Gagnon and Ansel 2019). Among others, miR-146a is a well-characterized miRNA taking part in multiple pro-inflammatory pathways (Boldin et al. 2011; Stickel et al. 2014, 2017). Functionally, miR-146a regulates the immune response and controls excessive inflammation, mainly through the repression of the NK-κB pathway (Taganov et al. 2006) and the polarization of macrophages towards the immunosuppressive, M2-type (Li et al. 2015). miR-146a displays multiple roles in the modulation of different pathways in different cancer types. According to the Kaplan–Meier plotter database (Nagy et al. 2018, 2021), a higher expression level of miR-146a is associated with a shorter overall survival (OS) in renal clear cell carcinoma (*p* = 0.04) and in hepatocellular carcinoma (*p* = 0.032) and with longer OS in bladder carcinoma (*p* = 0.0041), head-neck squamous cell carcinoma (*p* = 0.00044), breast cancer (*p* = 0.038), ovarian cancer (*p* = 0.0036) and lung adenocarcinoma (*p* = 0.012). However its expression level in plasma and the corresponding prognostic value remains to be clarified in HCC. The aim of this study was to investigate if miR-146a level might serve as prognostic factor in metastatic liver cancer of gastrointestinal origin undergoing sequential ablative therapy. Moreover, the relationship between miR-146a expression levels in plasma and immune infiltration of distant untreated metastatic TIME was analysed.

## Materials and methods

### Ethics approval and consent to participate

The study was approved by the local Ethics Commission (LMU München, Munich, Germany) and listed in the German clinical trials register (DRKS 00009744). All investigations were conducted in accordance with the Declaration of Helsinki. Before entering the study, all participants gave written informed consent.

### Study population and study design

In total, 21 patients with liver metastases of gastrointestinal cancer (*n* = 19, colorectal cancer; *n* = 2, pancreatic ductal adenocarcinoma) were included in this study. Patients were recruited between January 2018 and July 2021 at a tertiary care university hospital. Eligibility criteria included metastatic liver cancer involving both lobes with more than five lesions, absence of chemotherapy or cortisone treatment up until two weeks before study inclusion and no previous immunotherapy. Patients presenting with primary liver cancer and greater than 70% of liver involvement were excluded. The study protocol included: (1) baseline blood draw 24 h previous to unilobar ^90^Y-RE; (2) blood draw 48 h post-treatment; and (3) biopsy of the yet untreated, abscopal tumor in the contralateral liver lobe (median, 21 days, range 2–51). Immediately after the core biopsy, contralateral liver metastases underwent tumor ablation for completion of the therapeutic strategy. Clinical follow-up included contrast-enhanced MRI and/or CT every 3 months.

### Total RNA extraction

Peripheral blood was obtained 24 h before (T1) and 48 h after (T2) ^90^Y-RE. Blood (5 mL) was collected in EDTA tubes (Sarstedt AG, Nümbrecht, Germany) and centrifuged within 1 h from collection (1300 g, 5 min, 4 °C). Total RNA was isolated using the MagMAX™ *mir*Vana™ Total RNA Isolation Kit (ThermoFisher Scientific, Darmstadt, Germany; Supplementary Information).

### cDNA synthesis and quantitative real-time polymerase chain reaction (RT-qPCR)

For reverse transcription of miRNAs the TaqMan Advanced miRNA cDNA Synthesis Kit was used (ThermoFisher Scientific) and miRNAs were quantified using the TaqMan® Advanced miRNA Assay (ThermoFisher Scientific; Supplementary Information).

### Patient response assessment

To evaluate radiological response MRI abdomen was performed at baseline, before the second therapy and every 3 months follow up, supplemented by CT thorax/abdomen at baseline and at 6 months follow up. Images were evaluated according to the revised Response Evaluation Criteria in Solid Tumors (RECIST 1.1) (Eisenhauer et al. 2009) by two board-certified radiologists who were blinded to the clinical information and laboratory results. Patients were grouped as responders if they showed complete response (CR) or partial response (PR) at any time during follow-up. Vice versa, according to RECIST 1.1, patients were grouped as non-responders if they showed stable disease (SD, equals disease control) or progressive disease (PD) at any time during follow-up. Responder stratification was done in consensus between the two readers, in case of lack of agreement adjudication was made by a third radiologist.

### Histological and immunohistological analysis of tissue samples

Tissue samples were fixed in 10% formalin overnight at 4 °C and subsequently embedded in paraffin. Tissue sections’ preparation and immunohistochemical analysis are described in Supplementary Information.

### Multiplex immunophenotyping (mIF)

Immunophenotyping of paraffin sections was performed using the Opal 7 Tumor Infiltrating Lymphocyte kit (Akoya Biosciences, Marlborough MA, USA) for the detection of CD4 + , CD8 + and FoxP3 + T cells, according to the recommended protocol (Supplementary Information).

### Statistical analysis

Patient characteristics and survival data were compared with high vs. low miR-146a expression levels based on the mean value of 0.61 (pre RE) and 0.51 (post-RE), respectively. Patients were divided into group 1 (G1) and group 2 (G2): G1 included patients with an increase of miR-146a plasma levels at T2, while G2 included patients with a decrease of miR-146a plasma level at T2. For the comparison of high/low miR-146a groups, Fisher’s exact test was used for categorical data, Mann–Whitney U test was used for continuous data and Wilcoxon signed-rank test was used for paired continuous data. Progression-free survival (PFS) and overall survival (OS) were analyzed using the Kaplan–Meier method with log-rank testing. The OS was calculated from the date of the radioembolization session. The potential of miR-146a as a prognostic marker was calculated using the univariable Cox regression analysis. miR-146a levels were used as a continuous parameter. Correlation between the level of miR-146a and the clinical parameters and immune cell infiltration was explored using Pearson´s rank correlation tests. All tests were carried out two-sided. Variables’ distribution was evaluated with the Shapiro–Wilk test. Due to the low sample size, no alpha adjustment was made. All statistical tests were interpreted at a significance level of *α* = 5% with the according results considered exploratory. Statistical analyses were performed using SPSS Statistics 21.0.0 (IBM Corporation New York, NY, USA).

## Results

### Baseline characteristics

A total of 21 patients with liver metastatic CRC (*n* = 19, 90%) and PDAC (*n* = 2, 10%) were included in this study. The baseline characteristics are displayed in Table 1. The mean age of the patients was 63 years (range 32–83), 13 (62%) were male. At the time of recruitment, 4 patients presented extrahepatic metastases. Indication for radioembolization included non-response to second-line therapy (*n* = 17, 81.0%), non-response to first-line therapy (*n* = 2, 9.4%), contraindication to second-line therapy (*n* = 1, 4.8%), patient’s preference (*n* = 1, 4.8%). The median OS was 7.4 (range 2–40) months. Seven patients were lost to follow-up (at a median of 110 days). Response during follow-up was progressive disease (PD) in 14 (67%) patients, stable disease (SD) in 3 (14%) patients and partial response (PR) in 2 (9.5%) patients. Radiological images were not available for two patients, who were hence excluded from the response analysis.

**Table 1:** Baseline characteristics of patients

Variables	Total (%)	G1 (%)	G2 (%)
Patients	21	9 (43)	12 (57)
Age (years)			
Mean	63	63	63
Min–Max	32—83	53—83	32—78
< 65 (%)	10 (48)	6 (67)	4 (33)
≥ 65 (%)	11 (52)	3 (33)	8 (67)
Gender, *n* (%)			
Female	8 (38)	2 (22)	6 (50)
Male	13 (62)	7 (78)	6 (50)
Primary tumor	19 CRC (90)	9 (100)	10 (83)
	2 PDAC (10)	0	2 (17)
Fibrosis			
Yes (%)	1 (5)	1 (11)	0
No (%)	20 (95)	8 (89)	12 (100)
Maximum lesion, mm (%)			
≥ 50 (%)	7 (33)	4 (44)	3 (25)
< 50 (%)	14 (67)	5 (56)	9 (75)
Number of lesions (%)			
≥ 5 (%)	21 (100)	9 (100)	12 (100)
< 5 (%)	0	0	0
Extrahepatic metastases			
Yes	4 (19)	2 (9.5)	2 (2.5)
No	16 (76)	10 (48)	6 (28)
Unknown	1 (5)	0 (0)	0 (0)
Best response (%)			
PD (%)	14 (67)	5 (56)	9 (75)
SD (%)	3 (14)	2 (22)	1 (8.3)
PR (%)	2 (9.5)	1 (11)	1 (8.3)
Unknown (%)	2 (9.5)	1 (11)	1 (8.3)
Albumin (g/dL), mean ± SD	4.0 ± 0.39	4.2 ± 0.21	3.9 ± 0.45
Bilirubin (mg/dL), mean ± SD	0.76 ± 0.86	1.0 ± 1.26	0.6 ± 0.28
AST (U/L), mean ± SD	41.8 ± 16.42	46.89 ± 12.11	37.9 ± 18.60
ALT (U/L), mean ± SD	33.8 ± 17.19	40.89 ± 19.02	28.5 ± 14.22
GGT (U/L), mean ± SD	226.9 ± 192.41	199.11 ± 146.73	247.8 ± 224.82
ALP (U/L), mean ± SD	219.38 ± 160.74	200.0 ± 95.42	233.92 ± 199.55

Patients are displayed as total or grouped in G1 and G2 based on the miR-146a levels measured at T1 and T2. (G1: T1 < T2; G2: T1 > T2)

*ALP* alkaline phosphatase, *ALT* alanine transaminase, *AST* aspartate aminotransferase, *CRC* colorectal cancer, *G1* group 1, *G2* group 2, *GGT* gamma-glutamyl transferase, *LDH* lactate dehydrogenase, *PD* progressive disease, *PDAC*, pancreatic ductal adenocarcinoma; PR, partial response; SD, stable disease

### Changes in the plasma levels of miR-146a after ^90^Y-RE and association with TIME composition in untreated metastases

**Fig. 1:**
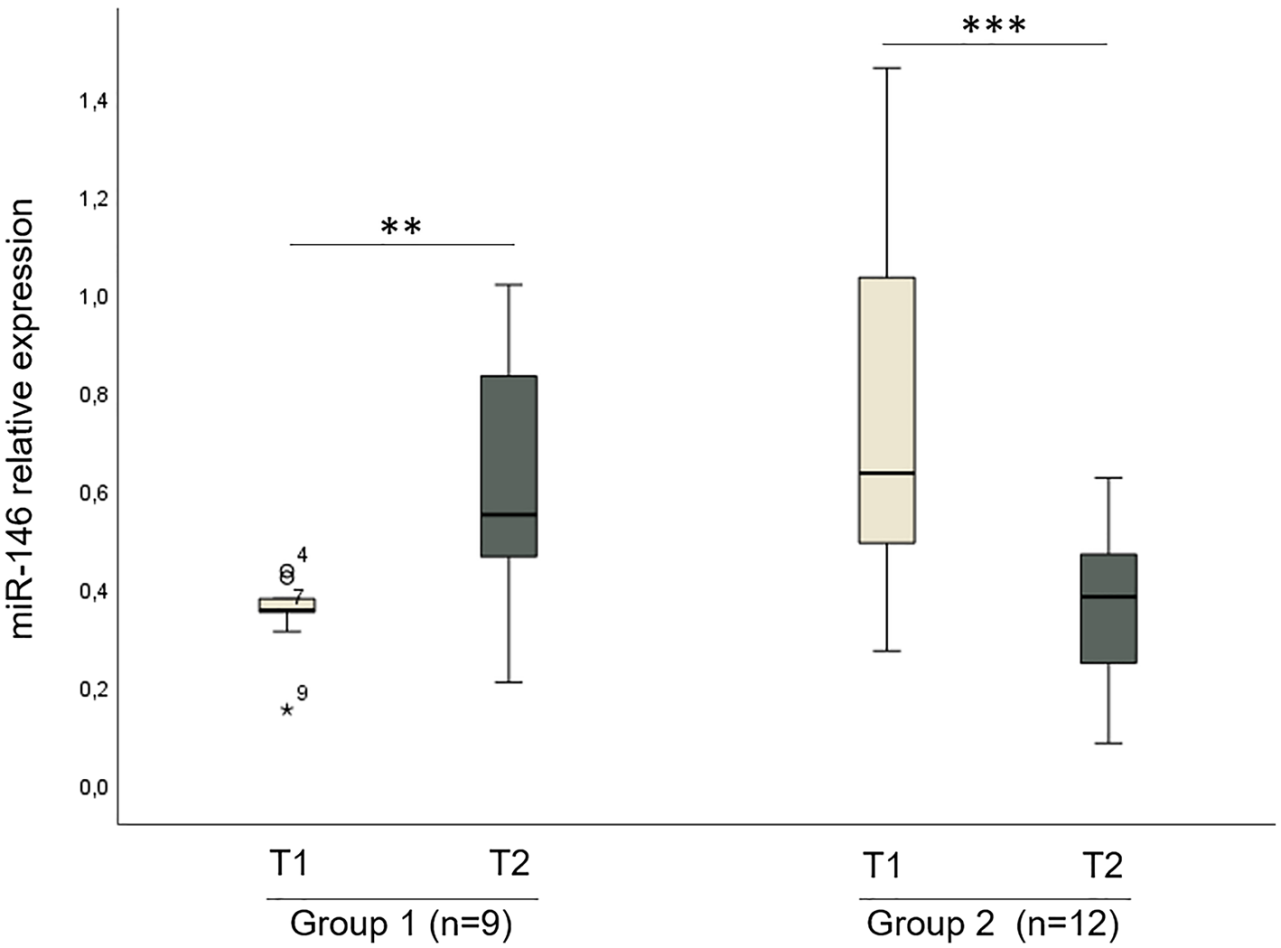
miRNA146a dynamics. Box plots represent the expression of miRNA-146a, analyzed by a single TaqMan assay, in plasma samples measured at T1 and T2. For a subgroup of 9 (43%) patients, miR-146a was significantly increased at T2 (*p* = 0.00048), while for remaining patients (*n* = 12, 57%), the level of miR-146a was significantly reduced (*p* = 0.004) at the same time point. Two patients were excluded since plasma was not available for both time points. Comparisons were performed using the Wilcoxon signed-rank test and the corresponding *p*-values of significant differences are indicated in the graphs (**p ≤ 0.01; ***p ≤ 0.001).Y axis represents the relative expression of miR-146a (measured as 2^−ΔCq^)

The comparison between the levels of miR-146a measured at T1 and T2 identified two distinct groups of patients with opposite trends: in group 1 (G1, *n* = 9, 43%) a significant increase (*p* = 0.00048) of miR-146a measured after therapy was observed, while group 2 (G2, *n* = 12, 57%) showed a significant decrease (*p* = 0.004) (Fig. 1).

Next, the correlation between the levels of miR-146a at T2 and TIME composition in untreated metastases was evaluated. The analysis included the quantification of CD4^+^ T cells (mean 20.64 ± 32.89 SD), CD8^+^ T cells (mean 240.98 ± 238.60 SD), FoxP3^+^ regulatory T cells (mean 4.00 ± 3.31 SD), and CD163^+^ M2 macrophages (mean 239.33 ± 100.55 SD). In addition, the level of expression of three exhaustion markers (PD1, PD-L1 and Tim3) was evaluated. No significant correlation between miR-146a levels and the absolute count of T cells, macrophages or PD1 + immune cells was found (all *p* > 0.05, Table 2). On the contrary, miR-146a measure at T2 positively correlated with the number of Tim3 + immune cells (*p* = 0.021) found in the TIME.

**Table 2:** Correlation analysis between the level of miR-146a at T2 and immune cell infiltration in metastatic lesion distant from therapy site. No positive PD-L1 cells were identified

Parameter	Rank correlation coefficient (*r*)	*p* value
CD4^+^	0.679	0.207
CD8^+^	-0.586	0.299
FoxP3^+^	0.388	0.518
M$\Phi$ (M2)	0.654	0.231
Tim3	0.654	0.021
PD-1	0.069	0.217
PD-L1	–	–

*M*$\Phi$ macrophages, *TIME* tumor immune microenvironment

**Fig. 2:**
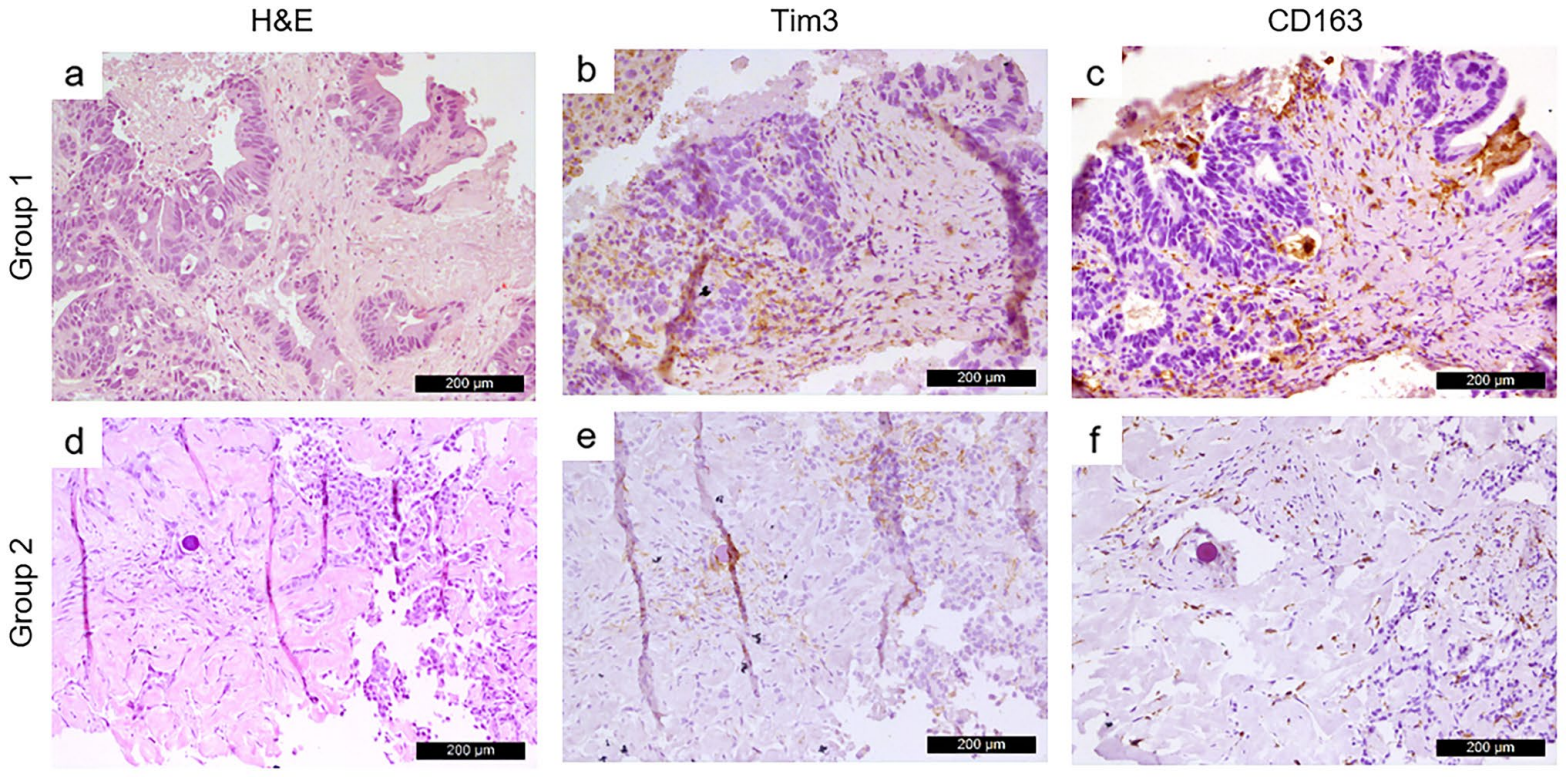
Representative images of Tim3 and CD163 immunohistochemical staining in mLC patients with high and low levels of miR-146a measured at T2. Patients from group 1 showed a higher level of Tim3 + (*p* = 0.036, panel **b**) and CD163 + (*p* = 0.423, panel **c**) infiltrating immune cells with respect to patients from group 2 (panel **e** and **f**, respectively). Hematoxylin and Eosin (H&E) staining is shown in panel **a** and **d**. Staining was performed on consecutive slides. Magnification: 200x. Scale bar: 200 µm

The comparison between intra-tumoral areas in the two patients’ groups revealed a significantly higher expression (*p* = 0.036) of Tim-3 in group 1 (Fig. 2b) with respect to group 2 (Fig. 2e). Similarly, tissues collected from group 1 showed a corresponding higher infiltration of CD163 + M2 macrophages (Fig. 2c) with respect to group 2 (Fig. 2f). However no statistical significance was found (*p* = 0.423).

**Fig. 3:**
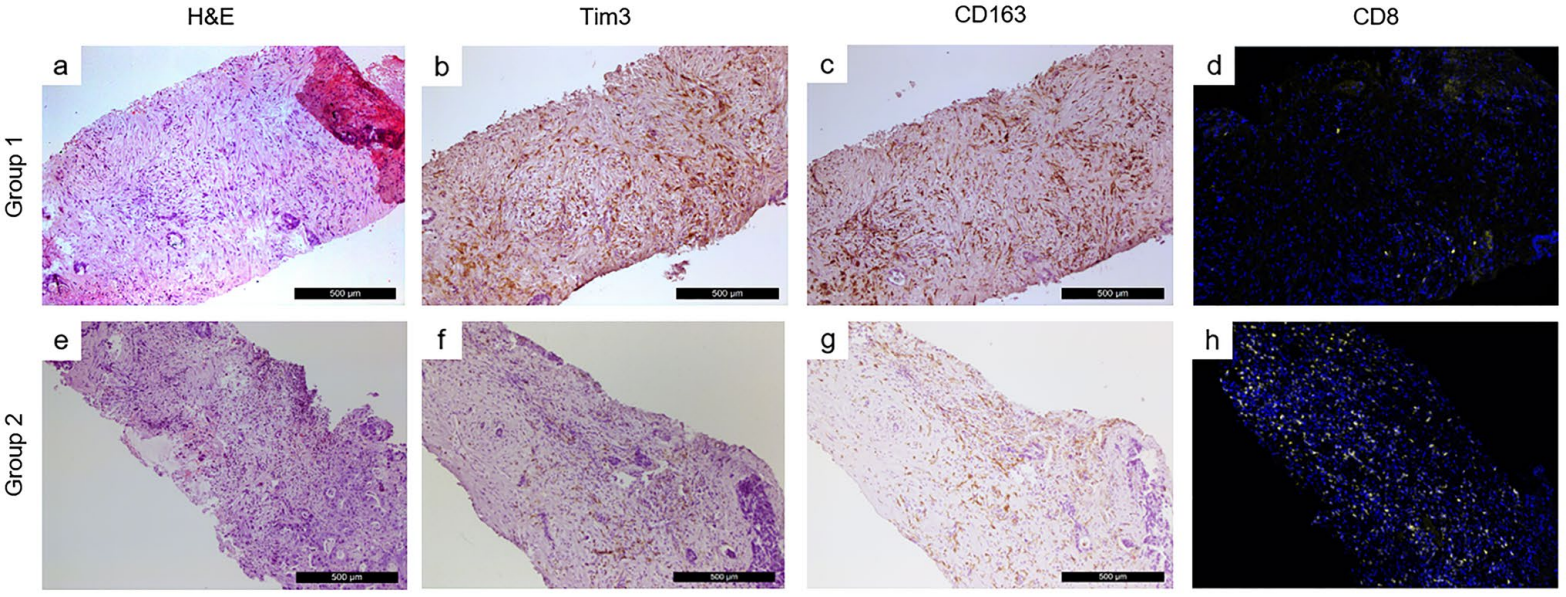
Representative images of Tim3 and CD163 immunohistochemical staining and CD8 immunofluorescence staining in mLC patients with high and low level of miR-146a measured at T2. In patients with higher levels of miR-146a measured after therapy (group 1), intratumoral areas were highly infiltrated by Tim3 + immune cells (panel **b**) and CD163 + M2 macrophages (panel **c**), while the level of CD8 + T cells infiltration was low (panel **d**). On the contrary, in patients with post-therapy lower levels of miR-146a (group 2), intratumoral areas showed marked infiltration of CD8 + T cells (panel **h**), while the infiltration of Tim3 + immune cells (panel **f**) and CD163 + M2 macrophages (panel **g**) was lower. Hematoxylin and Eosin (H&E) staining for G1 and G2 is shown in panel **a** and **e**, respectively. Staining was performed on consecutive slides. Magnification: 200x. Scale bar: 500 µm

To assess the potential role of Tim-3 in T cell exhaustion, the expression of Tim-3 was correlated with the number of CD4^+^, CD8^+^, and regulatory FoxP3^+^ T cells identified in the TIME. Immunohistological analysis of tissues collected from group 1 indicated a negative correlation between Tim3 + immune cells (Fig. 3b) and CD8 + T cells (Fig. 3d). Similar correlation was found between CD163 + (Fig. 3c) and CD8 + T cells. Opposite results were observed for group 2 (Fig. 3f–h). Additional IHC analysis of tissues collected from group 1 patients (n = 4) is shown in Supplementary Fig. 1.

### Association between miR-146a concentration, survival and therapy response

Univariable Cox regression analysis indicated a trend for shorter survival in patients with a higher level of miR-146a measured post ^90^Y-RE (*p* = 0.055, HR: 5.677, 95% CI: 0.963–33.451). With the exception of alkaline phosphatase, associated with shorter PFS (*p* = 0.029, HR: 1.004, 95%CI: 1.000–1.007), none of the remaining variables did show a significant association neither with OS nor PFS (all *p* > 0.05) (Table 3). Finally, no association between miR-146a levels, radiological response and survival endpoints measured by Kaplan–Meier analysis was found (data not shown).

**Table 3:** Univariable analysis of the variable associated with overall survival and progression-free survival in patients with mLC who underwent ^90^Y-RE

	OS Univariable analysis		PFS Univariable analysis	
Variable	HR (95% CI)	*p*-value	HR (95% CI)	*p*-value
Gender	0.472 (0.155–1.434)	0.185	0.771 (0.282–2.108)	0.613
Age (≥ 65 vs < 65)	0.923 (0.350–2.431)	0.870	0.786 (0.344–2.242)	0.786
Lesion size (≥ 5 cm vs < 5 cm)	0.378 (0.134–1.070)	0.067	1.124 (0.424–2.979)	0.814
miR-146a level (pre)	0.899 (0.324–2.492)	0.873	3.450 (0.174–72.038)	0.411
miR-146a level (post)	5.677 (0.963–33.451)	0.055	0.584 (0.081–4.235)	0.595
bilirubin	0.522 (0.099–2.742)	0.442	0.960 (0.555–1.660)	0.883
AST	1.021 (0.984–1.059)	0.272	0.996 (0.964–1.029)	0.814
ALT	1.022 (0.991–1.054)	0.166	1.000 (0.966–1.034)	0.983
GGT	1.001 (0.998–1.003)	0.531	1.003 (1.000–1.006)	0.065
ALP	1.000 (0.997–1.003)	0.814	1.004 (1.000–1.007)	0.029
Albumin	0.847 (0.252–2.844)	0.788	0.329 (0.070–1.549)	0.160

*ALP* alkaline phosphatase, *ALT* alanine transaminase, *AST* aspartate aminotransferase, *GGT* gamma-glutamyl transferase

### miR-146a levels do not correlate with liver function and damage

Next, the anti-inflammatory function of miR-146a was evaluated as a marker for liver parenchyma inflammation and damage induced by radiation. The level of miR-146a measured post ^90^Y-RE was correlated with the clinical parameters indicative of liver function (aspartate and alanine aminotransferase, bilirubin, albumin, alkaline phosphatase, gamma-glutamyl transferase), measured in a time frame between 1 and 3 months after ^90^Y-RE. Besides a trend towards a negative correlation between miR-146a levels and bilirubin (*r*: – 0.424, *p* = 0.055), miR-146a did not correlate to any other parameter of liver failure or tissue damage (Table 4).

**Table 4:** Correlation analysis between the level of miR-146a at T2 and the clinical parameters indicative of liver inflammation and liver function

	1 month		3 months	
Parameter	Rank correlation coefficient (r)	*p*-value	Rank correlation coefficient (r)	*p*-value
AST	– 0.267	0.381	– 0.070	0.820
ALT	– 0.265	0.246	– 0.053	0.856
GGT	– 0.104	0.654	0.038	0.898
Bilirubin	– 0.424	0.055	– 0.204	0.484
Albumin	0.081	0.728	– 0.193	0.527
ALP	– 0.159	0.490	– 0.011	0.971

*ALP* alkaline phosphatase, *ALT* alanine transaminase, *AST* aspartate aminotransferase, *GGT* gamma-glutamyl transferase

## Discussion

A growing body of evidence points to the role of ^90^Y-RE to stimulate an antitumor immune response, through the induction of cellular destruction and release of tumor-associated antigens (Chew et al. 2019). Nevertheless, ^90^Y-RE-induced, clinically measurable abscopal effects are rare (Powerski et al. 2020). Moreover, optimization of therapeutic strategies certainly requires the as of yet strong unmet medical need for predictive biomarkers. In the last decade, the discovery of minimally invasive, liquid biopsy-based biomarkers has shown large potential*.* Previous studies have addressed the effect of radiation on miRNA levels, as well as the correlation of specific miRNA with therapy response. In a lung cancer model, ionizing radiation induces the upregulation of miR-21 with inhibition of apoptosis (Wang et al. 2013), induction of cellular proliferation and therapy resistance (Wang et al. 2019a, b; Stechele et al. 2022). On the contrary, in a colorectal cancer model, the downregulation of miR-221 and miR-222 enhances radiation sensitivity (Zhang et al. 2011). Furthermore, in HCC and metastatic liver treated by local or locoregional therapies, the levels of miR-21, miR-210 and miR-122 increase and show predictive value (Andrasina et al. 2021).

In our own study, we aimed to investigate the significance of miR-146a as a non-invasive biomarker of TIME infiltration in distant metastases and its prognostic value. miR-146a did not correlate with any sign of deterioration in the liver function as a consequence of irradiation. Nevertheless, the univariable analysis indicated a trend toward an association between miR-146a and OS, while no association between miR-146a levels and therapy response was found. The histological TIME inspection after ^90^Y -RE showed an association of M2 macrophage infiltration with higher expression of Tim3 and a lower infiltration of CD8 + T cells in those patients with high levels of miR-146a. miR-146a belongs to a class of miRNA known as inflamma-miR, due to their multiple roles in inflammation. mR-146a inhibits the pro-inflammatory, anti-tumorigenic nuclear factor-κB (NF-κB) (Wang et al. 2019a, b) by targeting *IRAK1* and *TRAF6*, both proteins being part of the NF-κB pathway. miR-146a modulates also the macrophage M1–M2 polarization (Takeuchi and Akira 2010). M2 polarized macrophages mediate an anti-inflammatory and, therefore, pro-tumorigenic, response. In our study, immuno-histological analysis of distant metastases evidenced an increased M2 macrophage infiltration in those patients with high levels of miR-146a and low M2 macrophage infiltration in patients with low levels of miR-146a. This finding suggests that miR-146a can function as a time dependant biomarker of an anti-inflammatory, pro-tumorigenic status in liver metastases of GI cancer. Consequently, with further validation miR-146a might be considered an early marker for ^90^Y-RE efficacy: patients displayed benefit from ^90^Y -radioembolization when reduction of miR-146a levels were observed 48 h post therapy. The lack of correlation between survival and radiological response after ^90^Y -RE may likely be attributed to advanced disease stages in our cohort.

Furthermore, we did not find any direct correlation between plasma miR-146a and T-cell infiltration; however, we discovered a significant association between miR-146a and the exhaustion marker Tim-3 expressed by immune cells. Tim-3 demonstrated a trend to decrease if CD8 + T cells infiltration was increased in the same region. It should be noted that immunophenotyping presently does not address the co-expression of the respective markers. Co-staining of tissues with these markers will be necessary to confirm our results. Immune exhaustion is one of the hallmarks of cancer. Tim-3 is an immuno-regulator that negatively modulates T-cell response and T-cell apoptosis (Wolf et al. 2020; Sakaguchi et al. 1995; Sakuishi et al. 2010). miRNAs play a role in CD8^+^ T cell exhaustion, as demonstrated previously with miR-31 (Bhela and Rouse 2017) and miR-155 (Stelekati et al. 2018). We hypothesize that miR-146a might modulate the expression of Tim-3 in the TIME, leading to CD8^+^ T cell exhaustion. Future work will be necessary to characterize the CD8^+^ T cells present in the lesions. Our results suggest that patients displaying an increase of miR-146a after ^90^Y-RE may benefit from immunotherapy targeting Tim-3. Targeting miR-146a could be a novel strategy to improve the effect of radioembolization in patients receiving this type of therapy, alone or in combination with a downstream ablative therapy. Manipulation of miRNA levels by mimicking or inhibiting their expression represents an attractive strategy to sensitize tumours to radiation with a growing body of evidence supporting this approach (Chen et al. 2021). For example, the overexpression of tumor suppressor miRNAs such as miR-34a (Cortez et al. 2019) or members of the Let-7 family (Zhou et al. 2015) have been shown to sensitize lung and uveal melanoma tumor cells, respectively, to radiation. The downregulation of onco-miR such as miR-21 has been shown to arrest cell proliferation and angiogenesis (Tang et al. 2019). Accordingly, the downregulation of miR-146a in patients receiving radioembolization might therefore be a novel approach to improve therapeutic outcomes.

Limitations of this study must be acknowledged. The size of the cohort was small and inhomogeneous with respect to biology. Validation studies are necessary to confirm these preliminary data. miR analyses were conducted using core needle biopsies instead of larger resection material. For this reason, based on sampling error alone, the heterogeneity of the biomarker expression in the tumor may not be fully accurate. We were not able to define the origin of miR-146a, since the measurement of miR-146a was performed on plasma and we do not have paired PBMC available to exclude them as a major source. We do, however, intend to address this point in the future. Moreover, only a limited number of patients, tumor blocks collected before ^90^Y-RE were available. Finally, the time interval between ^90^Y-RE and sample acquisition was different throughout the study cohort.

In conclusion, miR-146a shows potential as a novel prognostic biomarker. A modulation of miR-146a levels in plasma might increase responses to ^90^Y-RE and could represent a supporting strategy in personalized therapy of metastatic liver.

## Supplementary Information

Below is the link to the electronic supplementary material.

Supplementary file1 (DOCX 3242 KB)

## Data Availability

The datasets generated during and/or analyzed during the current study are available from the corresponding author upon reasonable request.
