# Supplementary material for: Post-therapeutic microRNA-146a in liquid biopsies may determine prognosis in metastatic gastrointestinal cancer patients receiving 90Y-radioembolization
Source: J Cancer Res Clin Oncol. 2023 Jul 19;149(14):13017–26. doi: 10.1007/s00432-023-05185-0 (PMC10587196; doi:10.1007/s00432-023-05185-0)
Supplement: Supplementary file 1 — Supplementary file1 (DOCX 3242 KB) [file 432_2023_5185_MOESM1_ESM.docx]

**Supplementary Information**

**Post-therapeutic microRNA-146a in liquid biopsies may determine prognosis in patients with metastatic gastrointestinal cancer receiving**

**^90^Y-radioembolization**

**Journal name: Journal of Cancer Research and Clinical Oncology**

Heidrun Hirner-Eppeneder^1*^, PhD; Elif Öcal^1*^, MD; Matthias Stechele^1^, MD; Osman Öcal^1^, MD; Sijing Gu^1^, MD; Melanie Kimm^1^, PhD; Moritz Wildgruber^1^, MD; Lukas Salvermoser^1^; Philipp Kazmierczak^1^, MD; Stefanie Corradini^2^, MD; Martina Rudelius^3^, MD; Guido Piontek^3^; Maciej Pech^4^, MD; Shraga Nahum Goldberg^5^, MD; Jens Ricke^1^, MD; Marianna Alunni-Fabbroni^1#^, PhD.

^1^Department of Radiology, University Hospital, LMU Munich, Munich, Germany

^2^Department of Radiation Oncology, University Hospital, LMU Munich, Munich, Germany

^3^Department of Pathology, University Hospital, LMU Munich, Munich, Germany

^4^Department of Radiology and Nuclear Medicine, University of Magdeburg, Magdeburg, Germany

^5^Goldyne Savad Institute of Gene Therapy and Division of Image-guided Therapy and Interventional Oncology, Department of Radiology, Hadassah Hebrew University Medical Center, Jerusalem, Israel

^*^Equal contribution

^#^ Corresponding author

**Corresponding author**:

Marianna Alunni-Fabbroni

Marianna.Alunni@med.uni-muenchen.de

**Total RNA extraction**

Total RNA was isolated using the MagMAX^TM^ *mir*Vana^TM^ Total RNA Isolation Kit (ThermoFisher Scientific, Darmstadt, Germany) according to the manufacturer´s instructions. Briefly, plasma (100µl) was digested with Proteinase K and RNA purification was done using RNA binding beads and a magnet stand. After treatment of the samples with TURBO DNase^TM^ the RNA was eluted in 50µl of pre-heated Elution Buffer. The amount of RNA was determined on a Nanodrop spectrophotometer (Implen, Munich, Germany) and RNA was stored at -20°C.

**cDNA synthesis and quantitative real time polymerase chain reaction (RT-qPCR)**

For reverse transcription of miRNAs the TaqMan Advanced miRNA cDNA Synthesis Kit was used (ThermoFisher Scientific) and miRNAs were quantified using the TaqMan^®^ Advanced miRNA Assay (ThermoFisher Scientific) using the following primer assay purchased from ThermoFisher Scientific: hsa-miR-146a-5p (478399-mir), hsa-miR-16-5p (477860-mir), hsa-miR-19a-5p (479228-mir) and hsa-miR-26a-5p (477995-mir) (endogenous controls). Relative mRNA expression was calculated using the equation 2^-ΔCq^, where ΔCq = (Cq target mRNA)-(Cq reference mRNA) (Livak and Schmittgen 2001). Each primer was tested separately to define the PCR amplification efficiency using calibration curves. Correlation coefficient (r^2^) and PCR efficiency calculated from slope were all between 0.963 - 0.996 and 91 - 95%, respectively. All samples were run in triplicate; for each assay, no template controls were included to each plate. Plates were run on a Quant Studio 5 Fast Real-Time PCR System (ThermoFisher Scientific).

**Histological and immunohistological analysis of tissue samples**

Paraffin-embedded tissue sections including tumor and peri-tumor liver tissue were analyzed. Two μm thick tissue sections were deparaffinized, hydrated, boiled for antigen retrieval and blocked for endogenous peroxidase activity. Detection of primary antibodies (rabbit monoclonal anti-Tim-3, clone ERP22241, dilution 1:250; mouse monoclonal anti PD-1, dilution 1:50; (Abcam, Cambridge, UK) rabbit monoclonal anti-CD163, clone D6U1J, dilution 1:250 (Cell Signaling, Leiden, Netherlands) was done using Dako Envision + HRP Labeled Polymer system. The DAB substrate kit (Cell Signaling) was used as a chromogen. Sections were counterstained with hematoxylin, and mounted using Neo-Mount (Roth, Karlsruhe, Germany). For each biopsy, after low magnification scanning at × 10, 5 individual fields at higher magnification (× 40) were chosen randomly in order to capture various elements of tumor heterogeneity. Five microphotographs from each slide stained for the different markers were captured (2560 x 1920 pixels) with a Leica DM 2500 microscope (Leica, Wetzlar, Germany). The staining area of each marker was quantified by ImageJ software (Jensen 2013).Quantification was performed manually on the 5 fields by a board certified pathologist and a senior scientist.

**Multiplex immunophenotyping**

Immunophenotyping of paraffin sections was performed using the Opal 7 Tumor Infiltrating Lymphocyte kit (Akoya Biosciences, Marlborough MA, USA) according to the recommended protocol. Briefly, 2 μm tissue sections were deparaffinized, hydrated, boiled for antigen retrieval and blocked for endogenous peroxidase activity. For each antibody (anti-CD4, -CD8, -FoXP3), a complete staining cycle including blocking, primary antibody incubation, secondary antibody incubation, fluorochrome incubation, and antigen removal was performed. Slides were covered with VectaShield mounting medium (Vector Laboratories, Burlingame CA, USA) and image acquisition was performed using the VectraPolaris scanning system (Akoya Biosciences). A board certified pathologist and a senior scientist performed quantification of positive cells from the most representative areas.

**
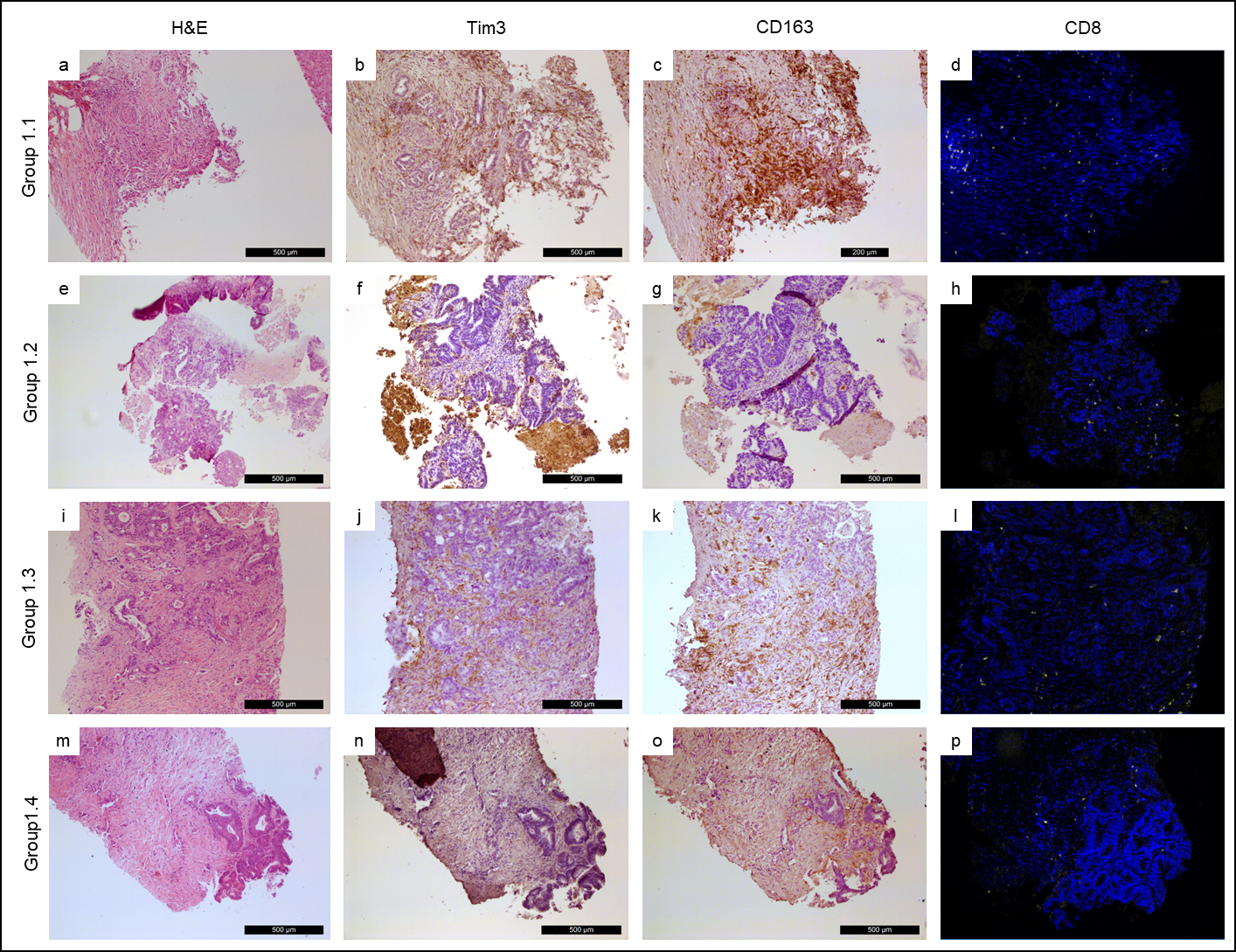
**

**Supplementary Fig. 1** H&E staining and immunohistochemical staining of Tim3, CD163 and CD8 from tissues collected from 4 patients belonging to the group 1 (Group1.1-1.4). Hematoxylin and Eosin (H&E) is shown in panel a, e, i and m, respectively. Staining was performed on consecutive slides. Magnification: 200x. Scale bar: 500 µm
